# Diminished ovarian reserve causes adverse ART outcomes attributed to effects on oxygen metabolism function in cumulus cells

**DOI:** 10.1186/s12864-023-09728-0

**Published:** 2023-10-31

**Authors:** Cong Zhang, Shi Song, Ming Yang, Liying Yan, Jie Qiao

**Affiliations:** 1 Center for Reproductive Medicine, Department of Obstetrics and Gynecology, Peking University Third Hospital, Beijing, 100191 China; 2 National Clinical Research Center for Obstetrics and Gynecology (Peking University Third Hospital), No. 49, North Garden Road, Haidian District, Beijing, 100191 China; 3 School of Basic Medicine (Hebei Medical University), Shijiazhuang, 050000 China; 4 Key Laboratory of Assisted Reproduction (Peking University), Ministry of Education, Beijing, 100191 China; 5 Beijing Key Laboratory of Reproductive Endocrinology and Assisted Reproductive Technology, Beijing, 100191 China; 6 Research Units of Comprehensive Diagnosis and Treatment of Oocyte Maturation Arrest (Chinese Academy of Medical Sciences), Beijing, 100191 China; 7 Savid Medical College (University of Chinese Academy of Sciences), Beijing, 100191 China

**Keywords:** Cumulus cell, Advanced age, Decreased ovarian reserve, Transcriptome, Assisted reproductive technology

## Abstract

**Background:**

Declining oocyte quality in women with advanced age has been a major impediment to assisted reproductive treatments’ (ART) success rate. However, aging is often accompanied by a diminished ovarian reserve (DOR). Cumulus cells (CCs) are known to play an important role in the development and maturation of oocytes, and the quality of CCs actually reflects the quality of the oocyte. In this study, CCs were used to investigate the real reasons for the decline in oocyte quality in older women.

**Methods:**

Ninety-nine CC samples were subdivided into 4 different groups according to the different age and ovarian reserve status. Other than clinical ART results, transcriptional expression profiles were performed in CCs to detect the differences.

**Results:**

The results were that DOR, no matter in young or advanced age group, was found to be significantly associated with adverse ART outcomes. Of note, there were no statistically significant changes in ART outcomes in the group at advanced age with normal ovarian reserve (NOR), compared to the young with NOR. DOR induced a series of transcriptional variations in CCs commonly enriched in oxygen metabolism.

**Conclusion:**

Our results revealed that the ART outcomes in advanced patients were attributable to the DOR. The oxygen metabolic changes may interfere with CCs’ function of supporting oocytes. This study can provide guidance for ART practice that not age but ovarian reserve status is the main predictor for ART outcomes, and ovarian reserve status should be timely assessed when the clinical manifestations are still mild in elderly women.

**Supplementary Information:**

The online version contains supplementary material available at 10.1186/s12864-023-09728-0.

## Introduction

In modern society, due to the demand of the labor market, the improvement of contraceptive means, the increase in life expectancy and the development of assisted reproductive technology (ART), more and more women choose to delay their reproductive age, but advanced age often leads to decreased fertility. According to the data of National Bureau of Statistics of China, the fertility rate decreased significantly in women age ≥ 35 years from 2003 to 2018 (http://www.stats.gov.cn/). In addition, advanced pregnancy does adversely affect women and their offspring, such as increasing the incidence of pregnancy complications and adverse pregnancy outcomes, and affecting the long-term health of newborns [1].

The decreased ovarian reserve (DOR) may contribute to the decrease of fertility in elderly women. DOR refers to not only the decrease of the number of follicles in the ovary, but also the quality of oocytes. The decreased oocyte quality of elderly women reflects in multiple aspects. For example, the abnormal meiotic process during oocyte maturation in elderly women leads to increased incidence of aneuploidy [2], abnormal mitochondrial respiratory chain in elderly oocytes leads to energy supply disorders [3], peroxide accumulation leads to DNA damage [4, 5], and asynchronous development of cytoplasm and nuclear leads to failure maturation [6]. Even if those oocytes continue abnormally developed and finally matured, they may still lead to fertilization failure or low embryonic developmental potential. Studies have shown that the pregnancy outcome of elderly women who received donated young oocytes after IVF treatment is significantly better than that of using their own oocytes [7]. However, we should note that advanced age is not completely equivalent to DOR. Even some young women can experience ovarian reserve failure in advance, which is known as premature ovarian failure (POF). For many women who are defined as advanced age, however, their may not present significant symptoms of decreased ovarian reserve. Therefore, our study aims to further investigate the relationship between the effect of increasing age and the performance in the state of DOR.

Microenvironment is essential for oocyte development, during which granulosa cells, around oocytes during follicular development, especially play an important role. Granulosa cells are a single layer of flat cells surrounding oocytes in primordial follicles. With primordial follicles developing into preovulatory follicles, granulosa cells gradually transform into multi-layered columnar cuboidal epithelium and differentiate into parietal granulosa cells and cumulus cells (CCs). In particular, CCs are in direct spatial contact with oocytes at the final stage of oocyte maturation, and studies have shown that there is an abundant and complex material and information exchange between CCs and oocytes [8]. Therefore, it is expected that CCs’ pathological conditions can help predict oocyte quality. Here, in this study, CCs at different ages and ovarian reserve status reveal the changes in the microenvironment of oocyte development.

With the development and application of single-cell sequencing technology, researchers can re-understand the changes of specific cell groups in abnormal states from the perspective of single-cell or genomics, and may timely detect the changes at the cellular gene level when the clinical manifestations seem mild, making it possible to carry out active clinical intervention or indicate the prognosis as soon as possible. At present, through sequencing technology, researchers reach a certain understanding of the genomic changes of CCs caused by aging. For example, Wang et al. [9] firstly revealed the transcriptome expression profile of different cells in the ovary, including granulosa cells, during aging in non-human primates. Moreover, he proposed the correlation between aging and oxidative stress related signaling pathways. In addition, the sequencing studies of Hurwitz et al. [10], Al-Edani et al. [11], and Molinari et al. [12] all showed that advanced age mainly affect the quality of granulosa cells by influencing several biological processes, such as oxidative stress, hypoxia, and inflammatory response etc. These above studies, however, seem to ignore the impact of the presence of DOR in most elderly women, that is, their results may be affected by DOR rather than simply caused by age. And researches mainly screen and obtain some genetically pathogenic genes through genome sequencing in the population of young women with DOR, such as FMR1 [13]. A recent study also focused on the transcriptomic patterns of CCs under different ovarian reserve states and found that the altered expression of *IL1RL1*, *IL33*, *SFRP4* and *S1PR1* in CCs may be related to the pathogenesis of DOR, but it is also unfortunate that this study, on the other hand, failed to consider the effect of age on ovarian reserve status [14]. Studies that take both age and ovarian reserve status into account are still limited.

In this study, a total of 99 human CC samples from different patients were made use of and were divided into 4 separate groups according to ages and ovarian reserve status of patients (Young age + NOR (YN) = 25, Young age + DOR (YD) = 25, Advanced age + NOR (AN) = 25, Advanced age + DOR (AD) = 24). We compared the transcriptional expression levels in CCs among different groups, and analyzed those transcriptomic differences with a combination of clinical indicators. This study can help understand whether the effect of advanced age and DOR on the developmental microenvironment of oocytes is different, and whether they play different roles in affecting the CCs, which in return impairing oocyte quality, and it is expected to provide new guiding significance in clinical practice.

## Methods

### Information of enrolled patients

A total of 99 patients receiving the ART treatments were enrolled in this study, and they were assigned to the following four groups according to their age or ovarian reserve status: (i) YN group: patients’ age < 35 years old with NOR (AMH > 2 ng/ml, AFC 5 ~ 10)(*n* = 25); (ii) YD group: patients’ age < 35 years old with DOR (AMH < 1 ng/ml, AFC < 5) (*n* = 25); (iii) AN group: patients’ age ≥ 35 years old with NOR (AMH > 2 ng/ml, AFC 5 ~ 10) (*n* = 25); (iv) AD group: patients’ age ≥ 35 years old with DOR (AMH < 1 ng/ml, AFC < 5) (*n* = 24). Inclusion criteria included: tubal structural or functional problems, sperm quality problems or other sexual dysfunction in men, abnormal cervical function, abnormal endometrial function. Exclusion criteria included polycystic ovary syndrome (PCOS), previous ovarian surgery, ovarian cysts, ovarian teratoma, endometriosis, thyroid dysfunction, diabetes, and obesity. All patients had a body mass index within the normal/overweight range (22.13 ± 2.73). The induction protocol included: long protocol (*n* = 25), short protocol (*n* = 16) antagonist protocol (*n* = 58), and oocyte retrieval in natural cycles (*n* = 1). When a follicle reached 20 mm in diameter, the COCs would be aspirated by vaginal ultrasound aspiration needle 36 h after HCG injection.

### Acquisition and RNA extraction of CC

The fresh donated COCs collected from the IVF laboratory were first observed under a stereomicroscope and then washed in PBS buffer to remove blood contaminants. The well-separated single-cell CCs were obtained after being digested in hyaluronidase for 5—10 min and then were washed twice in PBS to ensure that no blood cells remained in the CCs. 20–30 CCs were randomly selected for each sample and rapidly transferred to the well-configured RNA extraction lysis through a mouth pipette. RNA extraction lysis buffer was previously configured, including RNase Inhibitor (Thermo Fisher Scientific), triton X-100 (Merck, Sigma-Aldrich), barcode primer (New England Biolabs), dNTP ( Thermo Fisher Scientific), external RNA controls consortium (ERCC) (Thermo Fisher Scientific), nuclease-free water. Tubes containing lysis buffer with cumulus cells votex for 40 s, and following, cells were incubated at 72 °C for 3 min to fully lysed, after which the tubes were quickly transferred to ice and reverse transcription experiments were immediately performed.

### Single-cell libraries construction

Reverse transcription was performed on the mRNA in cell lysis, followed by 20—30 cycles of PCR amplification to obtain cDNA. The amplified full-length cDNA was fragmented to 200—300 bp and then purified, followed by end-repair and adapter ligation. Finally, the construction of the cDNA library was completed by adding Index tags and undergoing 15—20 cycles of PCR. The cDNA library quality was tested using GAPDH as a characterization cell quality marker gene. Single-cell RNA-seq libraries were constructed by NEB kit.

### RNA-seq data processing

All samples were sequenced using the Illumina—NovaSeq sequencing platform at a sequencing depth of 2G. Raw paired-end reads were screened via trim_galore (version 0.6.6) with default parameters. Next, the cleaned reads were aligned to the gencode human hg38 reference (release 32) using STAR aligner [15] with default settings. Further transcription quantification was conducted via feature-counts [16] with the corresponding gene annotation file from the gencode database. The transcript per million (TPM) value was calculated via in-house R scripts. All detected genes were used for downstream analysis and genes that were expressed in less than three samples were filtered out. Samples with less than 2000 expressed genes were discarded. In total, 99 CC samples passed the filtration standard.

### DEGs analysis

The command ‘findMarkers’ in R package ‘Seurat’ were used to conduct DEGs analysis with default parameters (‘slot = scale.data’, ‘test.use = wilcox’).

### GO/KEGG analysis

The “enrichGO” and “enrichKEGG” commands in the R package ‘clusterProfiler’ [17] were used to annotate the differentially expressed genes based on the GO and KEGG database, and the annotation results were used for further visualization in R.

### Validation of RNA-seq data by real time-polymerase chain reaction (RT-PCR)

A selection of genes displaying a statistically significant difference of expression (displaying a fold-change >  ± 0.5) between different group cohort was validated with RT-PCR. We collected CCs from 4 samples in YN, 4 samples in YD, 4 samples in AN, and 4 samples in AD by human peripheral blood lymphocyte separation solution (Tianjin haoyang Biological Manufacture Co.), and total RNA was extracted from CCs by Trizol method [18]. After RNA extraction was completed, the concentration was measured, and according to the requirement of PrimeScriptTM RT reagent Kit with gDNA Eraser (Takara Biomedical Technology Co.) that the total amount of RNA in the 20 μl amplification system should not be more than 1 μg, the concentration of the samples we obtained was uniformly diluted to 500 ng/μl and added 1 μl to the reverse transcription system.The primers used for RT-PCR were designed using tools from Integrated DNA Technologies (https://sg.idtdna.com/). Primer sequences are provided in Table 1. Real-time PCR was performed using PowerUp™ SYBR™ Green Master Mix and the QuantStudio 3 (appliedbiosystems by Thermo Fisher Scientific Co.). The PCR conditions used for all genes were as follows: denaturing cycle for 20 s at 95 °C; 40 PCR cycles (denaturing, 95 °C for 5 s, annealing and extension, 60 °C for 30 s), a melting curve (95 °C for 1 s, 60 °C for 20 s), and a final step cycle at 95 °C for 1 s. Each sample was performed for three replicate experiments and was analyzed in QuantStudio™ Design & Analysis Software. *ACTB* was chosen as a reference gene. Gene expression quantification was performed according to the 2^−ΔΔCt^ method. Analysis and presentation of results used GraphPad Prism 8.

**Table 1:** Primers used for RT-PCR

RefSeq transcript ID	Gene symbol	Sequence (5’ to 3’)	Amplicon size (bp)
NM_001101.5	*ACTB*	S: ACCTTCTACAATGAGCTGCG A: CCTGGATAGCAACGTACATGG	148
NM_001136.5	*AGER*	S: GAGTCCGTGTCTACCAGATTC A: ATCCAAGTGCCAGCTAAGAG	140
NM_001144999.3	*ITGAV*	S: AGAATCAAGGAGAAGGTGCC A: GGCGAGTTTGGTTTTCTGTC	138
NM_000244.4	*MEN1*	S: CACATCCAGTCCCTCTTCAG A: CAGGCATGATCCTCAGACAG	149
NM_032199.3	*ARID5B*	S: CGGCAACTTTTATCCAGCTC A: GAATGTACCCACTTGACCAGG	146
NM_001113182.3	*BRD2*	S: TTCTGGCTTTGGACCTTCTG A: TTCTCATCGTAACTCATGGGC	135
NM_001276270.2	*MBD4*	S: TCTGCTCAGTTTGGTGCTAC A: TGAACTTCAGTCCTTGTGGG	145
NM_199461.4	*NANOS1*	S: CTACACCACCCATATCCTCAAG A: GGGCAGTACTTGATGGTGTG	120
NM_003068.5	*SNAI2*	S: AGCATTTCAACGCCTCCA A: GGATCTCTGGTTGTGGTATGAC	117
NM_001145155.2	*NR2F2*	S: GCCATAGTCCTGTTCACCTC A: GGTACTGGCTCCTAACGTATTC	112
NM_021205.6	*RHOU*	S: GTACATCCCTACTGCCTTCG A: AGGAAGATGTCTGTGTTGGTG	144
NM_002184.4	*IL6ST*	S: GCAACATTCTTACATTCGGACAG A: TCCCACTCACACCTCATTTTC	133
NM_001077710.3	*FAM110C*	S: GAGCTGATAGAGCAGGTGC A: AGGTGTACAGCCACTTGATG	76
NM_001080529.3	*WIPF3*	S: GTAAGCACAGACACCTCCAG A: CCTTTAGAACTCTCGATCTGCG	143
NM_005532	*IFI27*	S: TCTGCCGTAGTTTTGCCC A: ATCATCTTGGCTGCTATGGAG	134
NM_002727.4	*SRGN*	S: CCTCATCCTGGTTCTGGAATC A: TGTTGGATTCACCTGGAAGTAG	149

*ACTB* Served as internal control, *S* Sense, *A* Antisense

### Code availability

The in-house Shell, Python, perl or R codes based on published software as described in the Methods are available upon request from the corresponding authors.

## Results

### Compared with YN, AN did not significantly cause changes in ART treatment outcomes, but there are changes in gene functions in CCs associated with inflammatory metabolism and cell cycle

**Fig. 1:**
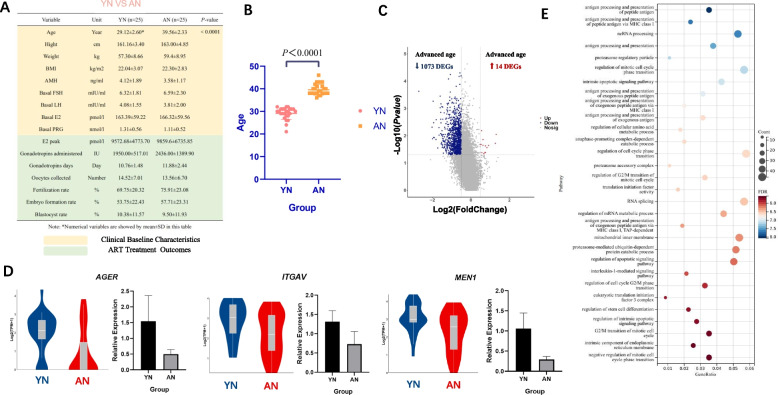
**A** Comparison of clinical baseline characteristics and ART cycle outcomes between YN and AN. The two groups are characterized by the different age and ovarian reserve thresholds: (i) YN: age < 35y, and NOR: AMH > 2 ng/ml, AFC 5 ~ 10; (ii) AN: age ≥ 35y, and NOR: AMH > 2 ng/ml, AFC 5 ~ 10. **B** Difference plot of YN vs AN in Age. **C** Differential analysis of CC transcriptomic data between YN and AN showed that 14 differential genes were up-regulated and 1073 differential genes were down-regulated in advanced age (*P*-value < 0.05, | avg-diff |> 0.5). **D** Some differential genes were verified by RT-PCR. **E** GO/KEGG analysis of all DEGs between YN and AN (Items are ranked and selected the top 30 items according to -log10(*P*-value) from largest to smallest)

For baseline information, there was no significant difference between the YN group (age < 35y, and NOR: AMH > 2 ng/ml, AFC 5 ~ 10) and AN group (age ≥ 35y, and NOR: AMH > 2 ng/ml, AFC 5 ~ 10) except for age (*P* < 0.0001). In addition, ART treatment outcomes did not significantly show any difference between the two groups (Fig. 1A, B). However, when comparing the transcriptomic data between the two groups of CCs, a total of 1087 differentially expressed genes (DEGs) were screened out, such as *AGER*, *ITGAV*, *MEN1*, etc. (Supplementary files, Normal_Young-vs-Old (Fig. 1C)), with the cut-off value of *P*-value < 0.05, | avg-diff |> 0.5 (Fig. 1C). Such differential transcriptional expression pattern was also seen in some genes verified by RT-PCR (Fig. 1D, Supplementary Figure A). To further analyze those DEGs, functional enrichment analysis was performed, where GO/KEGG analysis revealed top 30 enriched biological pathways or processes ranked by -log10 (*P*-value) from largest to smallest correlation (Fig. 1E). It is obvious that those DEGs were associated with several inflammatory pathways with 7 biological items involving 'antigen pathway and signaling' and another item 'interleukin-1-mediated cycle presentation'. Besides, another 7 biological items in Fig. 1E were related to cell cycle, which indicated that cumulus cells may experience some changes in cell cycle regulation during the natural aging of the ovary. Other metabolic pathways, such as apoptosis, RNA editing, and proteasome activity, may also play a role in the ovary natural aging process (Fig. 1E).

### Compared with YD, AD similarly did not cause significant changes in ART clinical treatment outcomes, and the biological processes potentially affected may mainly involve RNA splicing

**Fig. 2:**
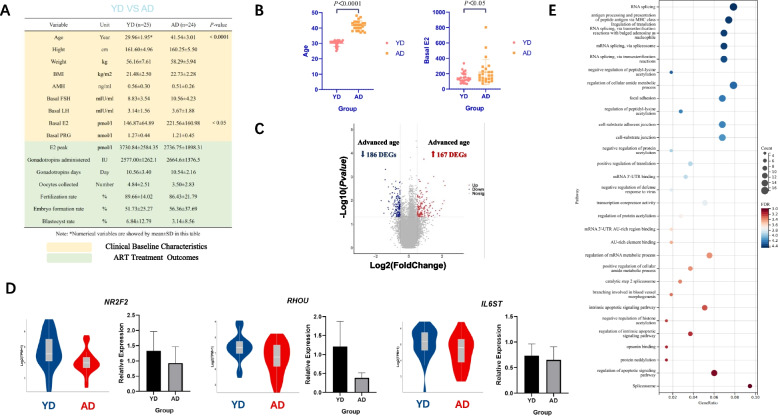
**A** Comparison of clinical baseline characteristics and ART cycle outcomes between YD and AD. The two groups are characterized by the different age and ovarian reserve thresholds: (i) YD: age < 35y, and DOR: AMH < 1 ng/ml, AFC < 5; (ii) AD: age ≥ 35y, and DOR: AMH < 1 ng/ml, AFC < 5. **B** Difference plot of YD vs AD in Age and Basal E2. **C** Differential analysis of CC transcriptomic data between YD and AD showed that 167 differential genes were up-regulated and 186 differential genes were down-regulated in advanced age (*P*-value < 0.05, | avg-diff |> 0.5). **D** Some differential genes were verified by RT-PCR. **E** GO/KEGG analysis of all DEGs between YD and AD (Items are ranked and selected the top 30 items according to -log10(*P*-value) from largest to smallest)

Comparison of basic clinical data between the YD group (age < 35y, and DOR: AMH < 1 ng/ml, AFC < 5) and AD group (age ≥ 35y, and DOR: AMH < 1 ng/ml, AFC < 5) revealed that in addition to the significant difference in age (*P* < 0.0001), basal estrogen level was significantly higher in elderly women with DOR (*P* < 0.05), but there was still no significant difference in ART treatment outcomes between the two groups (Fig. 2A, B). The transcriptome data of the two groups were also compared, and the differential genes were screened using *P*-value < 0.05, | avg-diff |> 0.5 as the standard. We obtained a total of 353 DEGs (Fig. 2C), such as *NR2F2*, *GREB1*, *IL6ST*, etc. (Supplementary files, Decrease_Young-vs-Old). Some genes were further verified by RT-PCR, which turned out to be the same expressed trend as the transcriptomics obtained by sequencing (Fig. 2D, Supplementary Figure B). GO/KEGG analysis was also performed on these DEGs. The enriched biological pathways involved 11 biological processes related to RNA cellular splicing function, 5 items related to intracellular acetylation function, 3 items related to apoptosis pathway regulation, 3 items were related to cell connection and adhesion, 2 items were related to translational regulation, and the remaining items were scattered in the regulation of metabolic responses that occurred in cells, such as the regulation of inflammatory response, vascular cell differentiation, opsonin binding and protein neddylation, which displayed a different pattern compared to enriched pathways between the two groups YN vs AN (Fig. 2E). This perhaps reflects that DOR as an intervening factor in aging may have an altered effect on the transcriptome expression profile of CCs with age increasing. However, it should be noted that the DOR with advanced age in this study was not completely equivalent to the situation of young women with DOR after increasing their age. Therefore, the comparison between the two groups may not fully explain the precise effect of age on the female population with DOR.

### Compared with YN, YD resulted in unfavorable ART clinical treatment outcomes, and these negative effects on CCs derived from a series of biochemical alterations evoked by hypoxic events

**Fig. 3:**
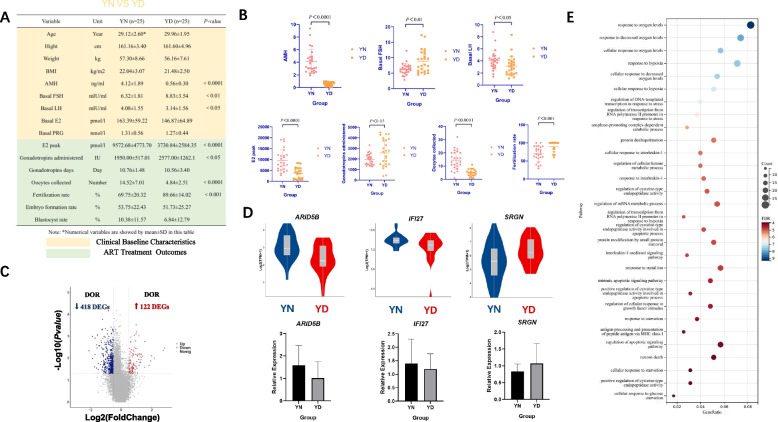
**A** Comparison of clinical baseline characteristics and ART cycle outcomes between YN and YD. The two groups are characterized by the different age and ovarian reserve thresholds: (i) YN: age < 35y, and NOR: AMH > 2 ng/ml, AFC 5 ~ 10; (ii) YD: age < 35y, and DOR: AMH < 1 ng/ml, AFC < 5. **B** Difference plot of YN vs YD in AMH, Basal FSH, Basal LH, E2 peak, Gonadotropins administered, Oocyte collected, and Fertility rate. **C** Differential analysis of CC transcriptomic data between YN and YD showed that 122 differential genes were up-regulated and 418 differential genes were down-regulated in DOR (*P*-value < 0.05, | avg-diff |> 0.5). **D** Some differential genes were verified by RT-PCR. **E** GO/KEGG analysis of all DEGs between YN and YD (Items are ranked and selected the top 30 items according to -log10(*P*-value) from largest to smallest)

Comparison of basic clinical data between YN group (age < 35y, and NOR: AMH > 2 ng/ml, AFC 5 ~ 10) and YD group (age < 35y, and DOR: AMH < 1 ng/ml, AFC < 5) revealed significant differences in basal FSH level and basal LH level (*P* < 0.01; *P* < 0.05) in addition to AMH (*P* < 0.0001), which indicated that DOR occurring in young women would affect basal reproductive endocrine hormone levels. The outcomes of ART treatment between two groups also showed statistical differences on multiple indicators, such as E2 peak (*P* < 0.0001), gonadotropins administered (*P* < 0.05), oocyte collected (*P* < 0.0001), and fertility rate (*P* < 0.01) (Fig. 3A, B). Similar to the differential expression analyses above, we obtained a total of 540 differential genes between the two groups (Fig. 3C), such as *ARID5B*, *IFI27*, *SRGN*, etc.(Supplementary files, Young_Normal-vs-Decrease). Some gene were verified by RT-PCR to have the same expressed trend as the transcriptomics obtained by sequencing (Fig. 3D). GO/KEGG analysis was performed on these DEGs. It can be easily noticed that seven closely enriched biological pathways were directly related to oxygen and hypoxic events, indicating possible underlying associations between oxygen metabolism and ovarian reserve status. Additionally, five items were related to apoptosis, four items were related to inflammatory response, three items were related to cellular glucose metabolism function, two items were related to intracellular stress response, and other biological function items included catabolic process, deubiquitination process, metabolic protein process, etc. (Fig. 3E). These DEGs-enriched entries may therefore reflect a series of linked negative effects resulting from DOR on CCs in the oocyte developmental microenvironment, and the dysfunction of CCs reflects the poor responsiveness of CCs to FSH secreted by the upstream pituitary gland in the axis of the female sex hormone regulation. In order to promote oocyte maturation, the required amount of FSH to be provided is increased, while the response to LH is also disturbed. Among the clinical outcomes, E2 peak was lower in the YD group. E2 levels on the day when patients were injected with LH analogues to promote oocyte maturation were known to actually reflect the number of oocytes, which was consistent with the reduction in oocyte collected number in DOR. The dosage of hormone therapy requiring ovulation induction in the YD group was significantly higher than that in the YN group, but at the same time, the number of collected oocytes obtained was significantly lower than that in the YN, which also confirmed the difficulty of ovulation induction in patients with DOR. However, we were surprised to find that the fertilization rate in the YD group was significantly higher than that in the YN group, but the embryo formation rate and blastocyst rate did not show significantly higher than those in the YN group, which indicated that the oocytes in the YD group did not show higher developmental potential.

### Compared with AN, AD also contributes to poor ART clinical outcomes, and negative effects on CCs may also be associated with hypoxic events and lipid metabolism

**Fig. 4:**
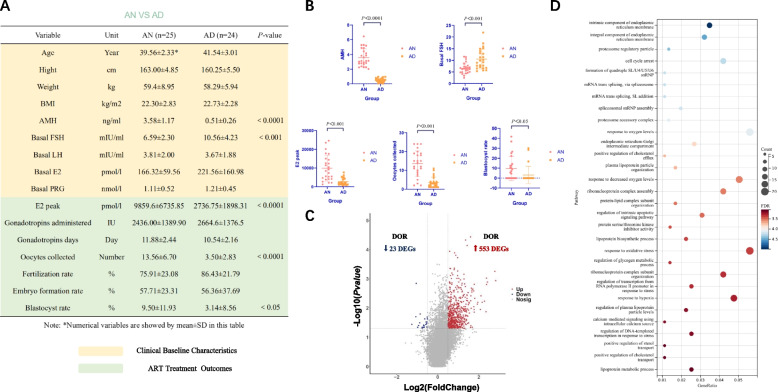
**A** Comparison of clinical baseline characteristics and ART cycle outcomes between AN and AD. The two groups are characterized by the different age and ovarian reserve thresholds: (i) AN: age ≥ 35y, and NOR: AMH > 2 ng/ml, AFC 5 ~ 10; (ii) AD: age ≥ 35y, and DOR: AMH < 1 ng/ml, AFC < 5. **B** Difference plot of AN vs AD in AMH, Basal FSH, E2 peak, Oocyte collected, and Blastocyst rate. **C** Differential analysis of CC transcriptomic data between AN and AD showed that 533 differential genes were up-regulated and 23 differential genes were down-regulated in DOR (*P*-value < 0.05, | avg-diff |> 0.5). **D** GO/KEGG analysis of all DEGs between AN and AD (Items are ranked and selected the top 30 items according to -log10(*P*-value) from largest to smallest)

Comparison of basic clinical data between AN group (age ≥ 35y, and NOR: AMH > 2 ng/ml, AFC 5 ~ 10) and AD group (age ≥ 35y, and DOR: AMH < 1 ng/ml, AFC < 5) revealed that in addition to significant differences in AMH (*P* < 0.0001), basal FSH levels were significantly higher in elderly women with DOR (*P* < 0.001). Comparing ART treatment outcomes between the two groups, it was found that there was a significant difference in E2 peak, oocyte collected, and blastocyst rate (*P* < 0.0001, *P* < 0.0001, *P* < 0.05) (Fig. 4A, B). The transcriptome data of CCs between the two groups were also compared. The differential genes were screened by *P*-value < 0.05, | avg-diff |> 0.5. We obtained a total of 576 differential genes (Fig. 4C), such as *SPRY2*, *ID1*, *CALR*, etc. (Supplementary files, Old_Normal-vs-Decrease). GO/KEGG analysis was also performed for these differential genes. The obtained enriched biological pathway items were ranked from largest to smallest according to -log10(*P*-value). The top 30 items were selected, of which 8 items were related to metabolism, which was related to the function of steroid hormone synthesis and secretion by CCs. 4 items were similar to the results of YN VS YD and related to intracellular oxygen content and hypoxia events. In addition, DOR affects other biological pathways in CCs of elderly women more abundantly, such as endoplasmic reticulum membrane function, proteasome function, mRNA editing and cell cycle (Fig. 4D). The effect of these differential genes and the cell biological processes affected on CCs is also reflected in ART treatment outcomes. The occurrence of DOR in young women leads to a significant decrease in both E2 peak and oocyte collected fertilization rate, while consistent results also occur in elderly women. Differently, the blastocyst rate decreased in the elderly female population with DOR compared with elderly women with NOR, but the fertilization rate and embryo formation rate did not show a significant difference. Similarly, we should also note that due to the effect of advanced age and DOR on the AD group, since the low number of oocytes retrieved may make the percentage standard deviation of embryo outcomes larger, and expanding the sample size may be the way to solve the problem.

### The intersection gene set of YN VS AN and YD VS AD illustrates that the function of commonly affected genes in the aging process mainly involves the metabolic process of nucleotides under different ovarian reserve states

**Fig. 5:**
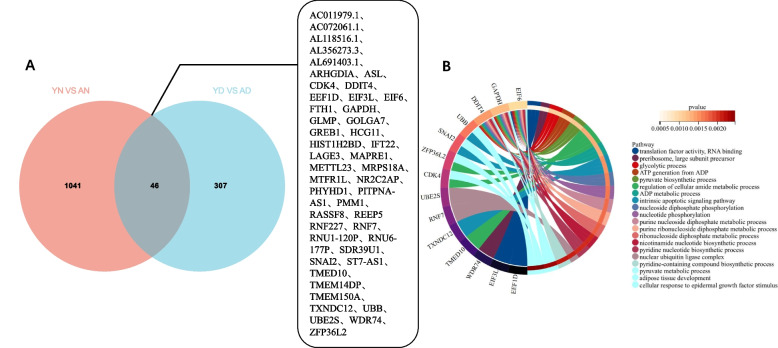
**A** The overlap co-DEG sets between YN vs AN and YD vs AD. **B** GO/KEGG analysis of all overlap co-DEGs (Items are ranked selected top 20 items according to -log10(*P*-value) from largest to smallest and all genes related to items are shown in pie chart)

In the above results, the differences between YN and AN illustrate the changes in gene level during natural aging, while the differences between YD and AD illustrate the changes in gene level during aging in the DOR state, but it may be possible that no matter in which group of comparisons, the number of differential genes obtained is higher under our existing threshold definition, and the categories of biological entries enriched by differential genes are widely distributed after GO/KEGG analysis, so we overlapped the differential genes obtained by comparing the two groups, and this gene set accurately reflects the effect of aging (whether natural or disease state) on CCs. Through overlap, we obtained a total of 46 differential genes (Fig. 5A), performed GO/KEGG analysis of these differential genes, ranked the correlations from largest to smallest by -log10 (*P*-value), and selected the top 20 items, and found that 7 pathways were associated with nucleotide-related metabolic activities. In addition, as many as 13 biological items involved in *DDIT4*/*EIF6*/*GAPDH* indicates that these three genes may play an important role in CC senescence (Fig. 5B).

### The intersection gene set of YN VS YD and AN VS AD illustrates that gene functions co-affected by DOR mainly involve mRNA function regulation at different age

**Fig. 6:**
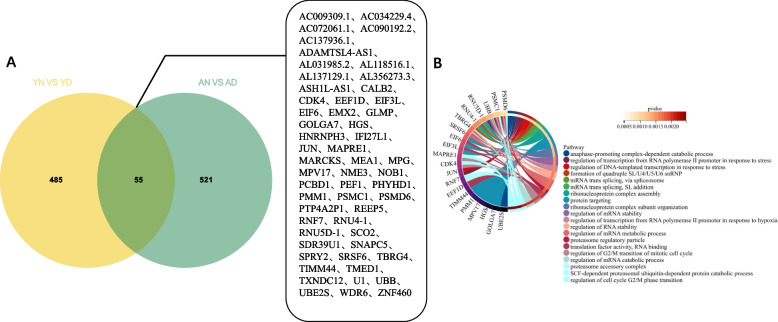
**A** The overlap co-DEG sets between YN vs YD and AN vs AD. **B** GO/KEGG analysis of all overlap co-DEGs (Items are ranked selected top 20 items according to -log10(*P*-value) from largest to smallest and all genes related to items are shown in pie chart)

In the above results, the difference between YN and YD indicated the effect of DOR on the gene level of CCs in young women, while the difference between AN and AD indicated the effect of DOR on the gene level of CCs in elderly women. However, no matter which group of comparison, the same as the aging comparison, under our existing threshold definition, the number of differential genes obtained is higher, and the biological item categories enriched by differential genes after GO/KEGG analysis are more widely distributed. Therefore, we also overlapped the differential genes obtained by the comparison of the two groups. These overlapped genes accurately reflected some common changes caused by the DOR in women of different ages. Through overlap, we obtained a total of 55 differential genes (Fig. 6A), performed GO/KEGG analysis of these differential genes, ranked the correlation according to -log10(*P*-value) from largest to smallest, and selected the top 20 items. Finally, we found that 10 items were related to the functional regulation of mRNAs, while others were scattered on ribonucleoprotein, proteasome, cell cycle, cell catabolism etc., and also found that PMSC1 and PSMD were involved in all 13 biological items, indicating that they were generally affected in CCs in the DOR state (Fig. 6B).

## Discussion

For elderly female population, a decrease in the number of follicles and a low developmental potential of oocytes are often observed whether in natural conception or ART. Recently, in studies focusing on the impact of age on oocyte quality, study designs tend to mix advanced patients and advanced patients with DOR together, which neglected the possible impact of DOR as an independent risk factor on the oocyte developmental microenvironment. Similarly, for the study of DOR, the age factor should also be taken into account separately. However, we should also be aware of the difference between POR and DOR in clinical treatment. POR refers to a pathological state in which the ovary responds poorly to gonadotropins [19], as evidenced by fewer follicles developing in the ovarian stimulation cycle, low peak blood oestrogens, high Gn dosage, a high rate of cycle cancellation, a low number of oocytes retrieved, and a low clinical pregnancy rate [20]. According to the POSEIDON criteria proposed in 2016 for the management of patients with POR [21], although the clinical characteristics of the YD and AD groups in this study fulfilled the criteria of III & IV (respectively: patients aged < 35 years with poor ovarian reserve indicators (AFC < 5, AMH < 1.2 μg/L); patients aged ≥ 35 years with poor ovarian reserve markers (AFC < 5, AMH < 1.2 μg/L)), but the AN group did not fulfil the criteria of II (patients aged ≥ 35 years with adequate ovarian reserve (AFC ≥ 5, AMH ≥ 1.2 μg/L) and unexpected poor or suboptimal ovarian response), which is what the present study aims to explore. Therefore, in this study, we try to clarify whether the effects of advanced age and DOR on oocyte development are separate or different, in hopes of providing new insights into therapeutic strategies for people with advanced age and DOR in clinical practice.

There has been considerable progress in studies on gene expression profiles of CCs focusing either on different ages or ovarian reserve status. In terms of the study of the effect of aging on CCs, the studies from Wang et al. [9], Al-Edani et al. [11], and Molinari et al. [12] compared the transcriptomic data between young and aged CCs, and found key differential expressed genes enriched in redox reactions and hypoxic events. Besides, Hurwitz et al. [10] found that some interesting genes involved in the inflammatory response witnessed changes in gene expression in aged CCs. Moreover, Kim et al. [22] proposed that age mainly affected intracellular steroidogenesis signaling pathways by comparing the CCs of young and elderly women who had a successful pregnancy through receiving ICSI. These studies above reasonably revealed the transcriptomic effect of advanced age on CCs while all fail to take into account that the role of DOR may play in influencing the risk factors of the oocyte developmental microenvironment in advanced age. Similarly, for research focusing on the effect of DOR on the transcriptomics of CCs, Chin et al. [23] and May-Panloup et al. [24] compared the gene alterations in CCs between NOR and DOR, while the study design did not control the age variable between the two groups, making age a confounding factor. Even if there was no difference in the comparative age between the two groups, the gene alterations in CCs may be different between DOR in young patients and DOR in the advanced age, which was obviously not reflected in the studies of Liu et al. [14].

Therefore, we here in further specifically grouped different ovarian reserve states at different ages, considering both factors in the hope of explaining the various effects of advanced age and DOR on CCs. Our results revealed that the effect of advanced age on ART pregnancy may be comparatively mild, and the ART outcome is not significantly worse whether it is in the NOR or DOR state. However, it cannot be ignored that there are actually transcriptional changes in some biological pathways in aged CCs, in which the inflammatory response-related pathways are mainly affected, consistent with the study of Hurwitz et al. [10]. On the other hand, at any age, DOR has an unfavorable effect on ART outcomes, and may impact the quality of CCs mainly by affecting the response pathways related to oxygen metabolism. For the advanced, this abnormal oxygen metabolism in CCs was confirmed by Wang et al. [9], Al-Edani et al. [11], and Molinari et al. [12] in previous studies. Moreover, in elderly women, DOR were found to bring about an effect on the lipid metabolism-related functions, partially consistent with the previous study [24, 25].

We then intersected two DEG sets that were screened from the two NOR groups and the two DOR groups, respectively. These overlapped 46 common DEGs were believed to be only affected by the age variable, and these genes were found to enrich in a more basal and active intracellular nucleotide metabolism pathway. Among these genes, for example, *DDIT4*, which has been suggested in previous studies to regulate cell growth, proliferation, and survival by inhibiting the activity of mTORC1 targets, plays an important role in the response to cellular energy levels and cellular stress, including responses to hypoxia and DNA damage [26, 27]. Similarly, the intersection of two DEG sets that were screened from the two young groups and the two advanced groups respectively was performed, and the overlapped 55 common DEGs were believed to be stably affected only by DOR. The enriched cellular function of these 55 common DEGs pointed to the regulation of intracellular basal functional mRNAs. It is easy to notice that in the previous part of our results, for different age groups (AD vs AN and YD vs YN), we observed that the DOR mainly affected the CCs transcriptional patterns via oxygen-related metabolic activity pathway, while the intersection DEGs of the two groups did not mainly enrich in this pathway. This may be because some overlapped genes were enriched in multiple similar biological processes due to the large coefficient of difference, such as *PSMC1* and *PSMD6*. *PSMC1* encodes a component of the 26S proteasome [28] and is involved in ubiquitinated protein-dependent degradation, playing a key role in maintaining proteostasis by removing misfolded or damaged proteins as well as proteins that no longer require function [29, 30]. *PSMD6* is also a component of the 26S proteasome [28] and involve in many cellular processes, including cell cycle progression, apoptosis, or DNA damage repair [31].

In summary, our study provides several new suggestions for clinical and basic experimental study. Firstly, not age but ovarian reserve status is the main predictor for ART outcomes. Therefore, that advanced age used to be an unfavorable independent predictor for ART outcomes needs to be re-evaluated. Clearly, our study showed that the elderly group with NOR did not show significant adverse ART outcomes compared to the young group. Age is a confounding factor for ART outcomes. Secondly, no matter at any age, DOR can lead to poor ART outcomes. This is possibly associated with effect of DOR on oxygen metabolism in CCs, and the decrease of CC quality may eventually lead to the impairment of oocyte quality. However, there are also some limitations in this study that the obtained CCs are luteinized CCs after LH stimulation, which may be different from CCs before LH stimulation. As most current epidemiological investigations do not differentiate between women with NOR in the older age groups, the results obtained are influenced by the factor of DOR, orienting the conclusions towards the age factor influencing the clinical outcomes [32–34], while research on natural ageing in female reproduction is still mostly focused on mechanistic [9, 19] and lacks data support from observational studies with large clinical sample sizes, and further validation in large samples is needed.

### Supplementary Information

**Additional file 1:** **Supplementary Figure.** **A** The gene relative expression in different groups by RNA-Seq. **B **The gene relative expression verified in different groups by RT-PCR. The file of DESCRIPTION contains a description of the names of the table data in the supplementary files.

## Data Availability

The raw sequencing data produced in this paper have been deposited in the Genome Sequence Archive in the BIG Data Center (accession ID: PRJCA007454), Chinese Academy of Sciences. All data is availiable at https://ngdc.cncb.ac.cn/gsa-human/ upon request.
