# Supplementary figures and images for: Diminished ovarian reserve causes adverse ART outcomes attributed to effects on oxygen metabolism function in cumulus cells
Source: BMC Genomics. 2023 Oct 31;24:655. doi: 10.1186/s12864-023-09728-0 (PMC10617226; doi:10.1186/s12864-023-09728-0)

Supplementary Figure

A

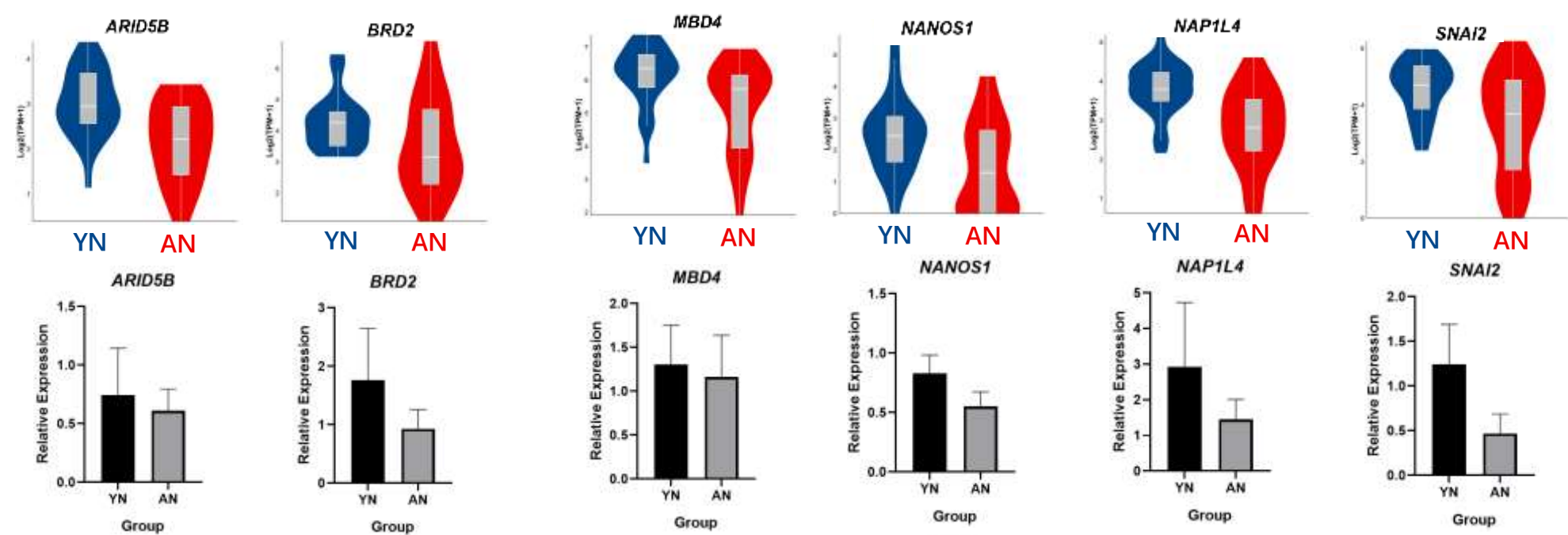

B

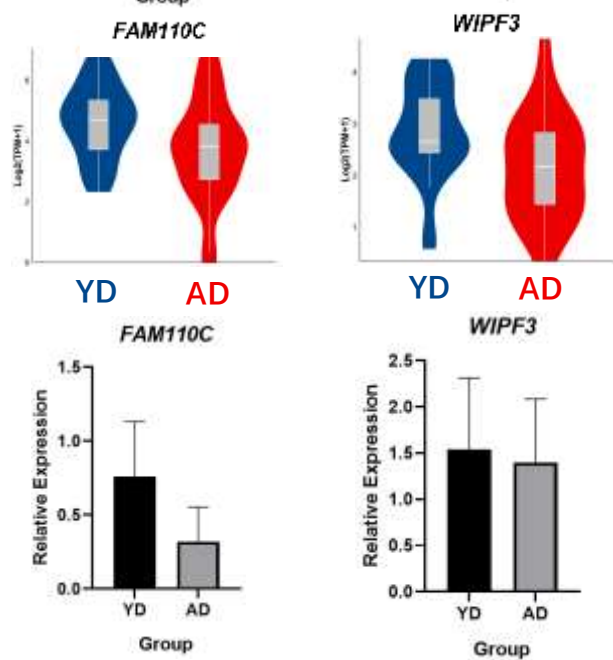

Supplement: Supplementary file 1 — Additional file 1: Supplementary Figure. A The gene relative expression in different groups by RNA-Seq. B The gene relative expression verified in different groups by RT-PCR. The file of DESCRIPTION contains a description of the names of the table data in the supplementary files. [file 12864_2023_9728_MOESM1_ESM.zip › Supplementary Figure.pdf]
